# Mitigation of Influenza B Epidemic with School Closures, Hong Kong, 2018

**DOI:** 10.3201/eid2411.180612

**Published:** 2018-11

**Authors:** Sheikh Taslim Ali, Benjamin J. Cowling, Eric H.Y. Lau, Vicky J. Fang, Gabriel M. Leung

**Affiliations:** The University of Hong Kong, Hong Kong, China

**Keywords:** influenza, school closure, public health, influenza B, Hong Kong, epidemic, China, intervention, transmission, viruses, respiratory infections, Yamagata, school outbreaks, outbreaks

## Abstract

In winter 2018, schools in Hong Kong were closed 1 week before the scheduled Chinese New Year holiday to mitigate an influenza B virus epidemic. The intervention occurred after the epidemic peak and reduced overall incidence by ≈4.2%. School-based vaccination programs should be implemented to more effectively reduce influenza illnesses.

Hong Kong, China, located on the coast south of Guangdong Province, has a subtropical climate and a population of 7.3 million. In Hong Kong, influenza epidemics occur during winter every year and sometimes during other seasons (*1*). One of the interventions that has been used by Hong Kong health authorities to control influenza epidemics is school closures; this intervention was previously applied in 2008 (*2*) and 2009 (*3*). During winter 2017–18, an epidemic of influenza B, Yamagata lineage, occurred in Hong Kong. The local media focused on this epidemic for 3 reasons. First, the occurrence of severe influenza cases in Hong Kong (*4*) attracted public concern. Second, the number of school outbreaks reported to the Centre for Health Protection in Hong Kong far exceeded the number reported in previous years (*4*). Third, a severe epidemic of influenza A(H3N2) was ongoing in the United States (*5*), which further increased local concern about influenza in general.

On February 7, 2018, the Hong Kong government announced that all 1,600 kindergartens, primary schools, and special needs schools in Hong Kong would close the following day, 1 week before the Chinese New Year school holiday, which in most schools was scheduled for February 15–23. Thus, in total, schools were closed for 2.5 weeks. We reviewed surveillance data on influenza and influenza-like illness (ILI) activity in Hong Kong to infer the effect of school closures on community transmission.

## The Study

As in previous studies, we used ILI surveillance data to indicate the incidence of influenza virus infections in the community (*1*,*6*,*7*). The Centre for Health Protection tracks a sentinel network of private doctors and reports the rates of outpatient consultations for ILI per 1,000 patient consultations every week (*4*), and the Public Health Laboratory Services branch reports the proportion of respiratory specimens testing positive for influenza virus by type and subtype every week (*8*). We multiplied the weekly ILI rates by the weekly influenza B virus detection rates to obtain a proxy (hereafter ILI+ proxy) measure of the number of cases of influenza B virus infection each week (Figure, panel A). We have previously shown that this ILI+ proxy provides an estimate that correlates linearly with the incidence of hospitalizations for influenza A(H1N1)pdm09 in Hong Kong (*6*); some have argued that this metric is a better linear correlate of the incidence of influenza illness than ILI rates alone or laboratory detection rates alone (*9*).

**Figure:**
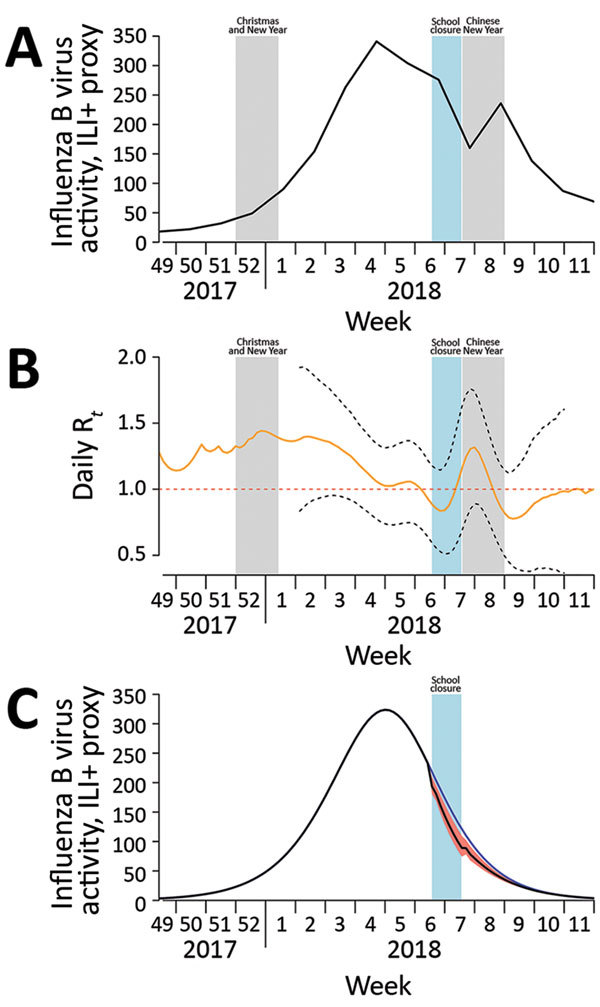
Influenza B virus activity, by epidemiologic week, Hong Kong, December 2017–March 2018. A) Incidence of influenza B virus measured by using the ILI+ proxy for influenza B, which is calculated by multiplying the weekly rate of ILI per 1,000 consultations by the weekly proportion of respiratory specimens submitted to the Public Health Laboratory Services (Hong Kong) that tested positive for influenza B virus (Technical Appendix Table 2). Shaded bars show school closure dates. B) Daily real-time estimate of transmissibility (R*_t_*) of influenza B virus. Black dashed lines indicate pointwise 95% CIs; red dashed line indicates transmission threshold. Shaded bars show school closure dates. C) Simulated incidence of influenza B virus with and without implementation of school closure (shaded bar) in Hong Kong during February 8–14, 2018. Blue line indicates the number of cases occurring during the hypothetical scenario of no school closure; black line indicates the number occurring with school closure, which reduced transmissibility by 16%. The difference between these 2 lines represents the 4.2% reduction in incidence of infections; red shading indicates 95% CI. R*_t_*, effective reproductive number at time *t*. ILI, influenza-like illness; R_t_, effective reproduction number at time *t*.

We calculated the ILI+ proxy for influenza B to infer the rate of person-to-person transmission of influenza B virus throughout the epidemic. We used the methods proposed by Cauchemez et al. (*10*) to estimate transmissibility by the effective reproduction number, R*_t_*, which represents the average number of secondary infections that result from a primary case of infection at time *t* (Technical Appendix). When R*_t_* exceeds 1, the epidemic is capable of spreading. We used flexible cubic splines to model the weekly influenza B ILI+ proxy values and interpolate daily ILI+ proxy values. We then estimated daily R*_t_* values from the daily influenza B ILI+ proxy values (*7*). We considered the serial interval distribution as the Weibull distribution, with a mean of 3.2 days and SD of 1.3 days (*11*). The estimated R*_t_* was 1.03 (95% CI 0.73–1.34) before the start of the school closure and 0.87 (95% CI 0.54–1.21) during the closure week, corresponding to a 16% (95% CI 10%–26%) reduction in transmissibility (Figure, panel B).

We then simulated the ILI+ proxy for influenza B under the counterfactual scenario of no school closures during February 8–14. Because R*_t_* is affected by the depletion of the susceptible population (*h_t_*, cumulative ILI+ proxy for influenza B at time *t*) and school closure (*C_t_*, indicator variable at time *t*), we first fitted a multivariable log-linear regression model for R*_t_* with *h_t_* and *C_t_* as explanatory variables (Technical Appendix). Using these estimated coefficients in a regression model, we then constructed the transmission rate (β*_t_*, function of initial transmission rate β_0_ and *C_t_*) for a susceptible-exposed-infected-recovered compartmental model to simulate incidence over time. To simulate incidence under no school closure, we set *C_t_* to 0 for the period February 8–14 (Technical Appendix).

Under the simulated epidemic with no school closures, the cumulative incidence for the entire epidemic was 0.527 (95% CI 0.472–0.574); the incidence was reduced to 0.505 (95% CI 0.494–0.519) when simulated with closures (Figure, panel C). This difference in proportions corresponds to a 4.2% (95% CI 1.5%–6.7%) reduction in infections from school closures. In sensitivity analyses, in which different levels of preexisting immunity (0.1%–30%) in the population were assumed, the estimated reduction in infections ranged from 3.3% (95% CI 1.2%–5.1%) for high (30%) preexisting immunity to 4.1% (95% CI 1.5%–6.7%) for low (0.1%) preexisting immunity. We also simulated the effect of school closures occurring 1 or 2 weeks earlier and estimated that infections would have been reduced by 8.6% if schools closed 1 week earlier (lasting for 2 weeks) and 13.5% if schools closed 2 weeks earlier (lasting for 3 weeks) (online Technical Appendix).

## Conclusions

In early 2018, schools were closed an extra week before a holiday in Hong Kong to mitigate an influenza epidemic. Closure after the epidemic peak had a small effect on transmission; we estimated a 4.2% reduction in overall incidence of infections. By the end of the 2017–18 winter season, ≈400 laboratory-confirmed influenza deaths had occurred among the local population of 7.2 million, lower than the rate in the contemporaneous influenza A(H3N2) epidemic in the United States but still a rate of moderate-to-high impact. A reduction in incidence of infections by 4.2% might have reduced hospitalizations and deaths by a similar percentage, with the caveat that hospitalizations and deaths would probably not have been equally distributed among age groups because most infections occur in children and most deaths in older adults.

The school closures were announced <24 hours before they began. We presume that the school closures were disruptive to parents’ schedules, potentially forcing some parents to stay home from work, and that many children stayed home during closures (*12*). The 16% reduction in transmission we estimated was lower than that estimated for the school closures that occurred in Hong Kong during June–July 2009 (25% reduction) (*3*). In 2009, the goal was to delay community transmission and spread out disease activity peak; thus, intervention before the peak was essential. In our study, R*_t_* appeared to increase (Figure, panel B) during the Chinese New Year, probably because of increased social interactions during holiday gatherings.

We assumed that the ILI+ proxy for influenza B was linearly correlated with the incidence of infections (*1*,*6*,*7*). This correlation could have been affected by changes in healthcare seeking behavior that might have resulted from private clinic closure, which occurred for a few days during the Chinese New Year holiday. This decreased healthcare access might have shifted the estimated reduction of influenza infections upward.

Influenza vaccination is considered the best preventive measure against influenza. However, >10 years after introduction of the influenza vaccination subsidy scheme for children, influenza vaccination coverage is still low in Hong Kong: ≈10% overall and ≈15% in children for the 2016–17 and 2017–18 winter seasons (*13*). To further increase influenza vaccination coverage in children, a school-based vaccination program should be implemented for the upcoming 2018–19 winter season.

Technical Appendix Description of methods, sensitivity analysis, and incidence of influenza B virus and influenza-like illnesses.
