# Supplementary material for: Mitigation of Influenza B Epidemic with School Closures, Hong Kong, 2018
Source: Emerg Infect Dis. 2018 Nov;24(11):2071–3. doi: 10.3201/eid2411.180612 (PMC6200008; doi:10.3201/eid2411.180612)
Supplement: Technical Appendix — Description of methods, sensitivity analysis, and incidence of influenza B virus and influenza-like illnesses. [file 18-0612-Techapp-s1.pdf]

Article DOI: https://doi.org/10.3201/eid2411.180612

Mitigation of Influenza B Epidemic with School Closures, Hong Kong, 2018

Technical Appendix

log-Linear Regression Model for Real-Time Effective Reproduction Number R_t

The real-time effective reproduction number at time t, denoted by R_t, represents an instantaneous measure of transmissibility, defined as the average number of secondary infections generated by a typical primary infectious case at time t. When R_t exceeds 1, the epidemic will continue to spread. R_0 is the basic reproduction number (a measure of initial transmissibility), S_t is the proportion of susceptible individuals at time t, and I_t is the number of infectious cases at time t. Based on standard general epidemic theory, R_t depends on the initial intensity of disease transmission (i.e., R_0) and the proportion of susceptible individuals at that time t (i.e., S_t), i.e., $R_t = R_0 S_t$. Hence, R_t is the same as R_0 if all individuals are susceptible ($S_t = 1$), which sometimes happens at the beginning of an epidemic especially for a novel disease. Under the same condition, R_t will decrease over time when the susceptible population (S_t) becomes depleted. In reality, R_t might be modified by other factors, such as introduction of control measures. Here, we hypothesized that school closure (C_t) reduced transmissibility. Where C_t is an indicator variable defined as 1 for days under school closure and 0 for days not under school closure. This variable is modeled by a multiplier $e^{\lambda C_t}$, where λ is a coefficient indicating the effect of school closure. A negative λ indicates that school closure reduces R_t. Therefore, R_t can be expressed as $R_t = R_0 S_t e^{\lambda C_t}$ (equation 1). Also, $S_t = S_0 - h_t = S_0(1 + \alpha h_t)$; where, S_0 is the initial proportion of susceptible persons in the population, α (equal to $-1/S_0$) is a constant, h_t (equal to $\sum_{x=1}^{t-1} I_x$) is a variable indicating the depletion of susceptible persons in the population at time t. Using Taylor series expansion, we have $S_t \approx S_0 e^{\alpha h_t}$, then substitute into equation 1 and get the formula, $R_t = R_0 S_0 e^{\alpha h_t} e^{\lambda C_t}$. Taking logarithms of both sides, we reduce the formula, $\ln(R_t) = \ln(R_0 S_0) + \alpha h_t + \lambda C_t$, and finally simplified as $\ln(R_t) = K + \alpha h_t + \lambda C_t$ (equation 2), where $K = \ln(R_0 S_0)$ and α and

λ are regression coefficients for the depletion of susceptible persons and school closure, respectively.

Simulation of Influenza Activity by Susceptible-Exposed-Infected-Recovered (SEIR) Transmission Model

We simulated influenza activity by using the standard SEIR transmission model. The sets of differential equations that define the transmission model are $\frac{dS}{dt} = -\beta_t SI$, $\frac{dE}{dt} = \beta_t SI - \sigma E$, $\frac{dI}{dt} = \sigma E - \gamma I$, and $\frac{d\mathrm{R}}{dt} = \gamma I$, where S, E, I, and R denote the proportion of susceptible, exposed, infectious, and recovered persons, respectively. β_t is the transmission rate per infective case, which is a function of the effective reproduction number (R_t) as shown later. The rate of becoming infectious, σ, was set to value 0.625/d and the recovery rate, γ, to 0.763/d. With these values, the generation interval in the SEIR model is 3.2 days, a value that is typical for influenza virus (*1*). We chose the initial condition such that there was about a 2-week gap between the influenza B virus activity (influenza-like illness+ proxy for influenza B virus) peak and the start of the school closure. The simulation was carried out on the basis of the SEIR model, where the influenza activity is assumed to be proportional to I.

Based on the theoretical results of the SEIR model and relation between the transmission rate (β_t) and R_t, we have $\mathrm{R}_t = \frac{\beta_t}{\gamma} S_t$. Therefore, $\beta_t = \frac{\mathrm{R}_t}{S_t}\gamma = \mathrm{R}_0 \gamma e^{\hat{\lambda} C_t}$ (after substituting R_t from equation 1) = $\beta_0 e^{\hat{\lambda} C_t}$ (because $\beta_0 = \mathrm{R}_0\gamma$, when $S_0 \approx 1$). Hence, $\beta_t = \hat{\beta}_0 \, e^{\hat{\lambda} C_t}$; therefore, $\hat{\beta}_0$ can be estimated once we estimate R_0.

Applying the multivariable log-linear regression model as described by equation 2, the regression coefficients ($\widehat{K}$, $\hat{\alpha}$, and $\hat{\lambda}$) can be estimated. The value of S_0 represents the preimmunity in the population (e.g., $S_0 = 1$ indicates no preimmunity in the population). We reported results in the main text on the basis of no preimmunity. A sensitivity analysis was performed assuming different levels of preimmunity in the population (Technical Appendix Table 1). The estimated reduction in influenza B virus infections was shown to be robust over a plausible range of preimmunities. To simulate incidence under no school closure, we set $C_t = 0$ throughout the original school closure period.

Sensitivity Analysis

From the regression analysis, we found $\hat{\lambda}$ = –0.12 (95% CI –0.19 to –0.04) and $\hat{K}$ = 0.345 (95% CI 0.302–0.388). By using formula $\hat{K} = \ln(R_0 S_0)$, we find $R_0 S_0 = e^{\hat{K}}$ = 1.412. We assumed an immunity of 0.1%–30.1% at the start of the epidemic (i.e., reduced initial susceptibility) and performed the simulation under each scenario. We found a similar reduction in the infection rate during the implementation of a 1-week school closure in Hong Kong (Technical Appendix Table 1). Further, we also simulated the hypothetical impact of school closures 1 week or 2 weeks earlier, for 2 or 3 weeks total, respectively, and estimated that these school closures would have reduced the total infections by 8.6% or 13.5%, respectively.

Data Source

We used data on influenza virus from the Public Health Laboratory Services branch of the Centre for Health Protection, Hong Kong, and influenza-like illness activity using the sentinel influenza-like illness surveillance conducted by the Centre for Health Protection. We retrieved the data from https://www.chp.gov.hk/en/statistics/data/10/641/642/2274.html and https://www.chp.gov.hk/en/static/24015.html (Technical Appendix Table 2).

References

1. Cowling BJ, Chan KH, Fang VJ, Lau LLH, So HC, Fung ROP, et al. Comparative epidemiology of pandemic and seasonal influenza A in households. N Engl J Med. 2010;362:2175–84. PubMed http://dx.doi.org/10.1056/NEJMoa0911530

Technical Appendix Table 1. Sensitivity analysis of population with different preexisting influenza virus B immunities*

Preexisting immunity, %	S_0	R_0 (95% CI)	% Reduction of infections (95% CI)	School closure timing after peak, d
0.1	0.999	1.41 (1.35–1.48)	4.15 (1.48–6.70)	13
5.1	0.949	1.49 (1.43–1.55)	4.01 (1.43–6.47)	13
10.1	0.899	1.57 (1.50–1.64)	3.88 (1.38–6.22)	14
15.1	0.849	1.66 (1.59–1.74)	3.73 (1.33–5.97)	14
20.1	0.799	1.77 (1.69–1.84)	3.58 (1.28–5.70)	15
25.1	0.749	1.88 (1.81–1.97)	3.42 (1.22–5.43)	15
30.1	0.699	2.02 (1.93–2.11)	3.25 (1.16–5.14)	16

S_0, proportion susceptible; **R_0**, basic reproductive number.

Technical Appendix Table 2. Incidence of influenza virus B–positive specimens and influenza-like illness, Hong Kong, October 2017–March 2018

Week	No. specimens tested	No. specimens influenza virus B–positive	GP ILI rate, per 1,000 consultations
2017 Oct 15–2017 Oct 21	3,653	41	43.7
2017 Oct 22–2017 Oct 28	3,348	50	40.1
2017 Oct 29–2017 Nov 4	4,171	62	37.8
2017 Nov 5–2017 Nov 11	3,891	54	43.8
2017 Nov 12–2017 Nov 18	3,780	48	39.5
2017 Nov 19–2017 Nov 25	3,667	58	43.2
2017 Nov 26–2017 Dec 2	3,792	69	35.4
2017 Dec 3–2017 Dec 9	3,882	85	41.4
2017 Dec 10–2017 Dec 16	4,094	104	42.4
2017 Dec 17–2017 Dec 23	4,085	172	38.5
2017 Dec 24–2017 Dec 30	4,518	343	32.2
2017 Dec 31–2018 Jan 6	4,692	528	39.8
2018 Jan 7–2018 Jan 13	5,105	681	57.8
2018 Jan 14–2018 Jan 20	7,176	1,346	70.0
2018 Jan 21–2018 Jan 27	7,562	1,681	76.8
2018 Jan 28–2018 Feb 3	7,399	1,492	75.4
2018 Feb 4–2018 Feb 10	8,350	1,614	71.3
2018 Feb 11–2018 Feb 17	6,322	1,391	36.4
2018 Feb 18–2018 Feb 24	9,638	1,978	57.4
2018 Feb 25–2018 Mar 3	7,363	1,064	47.9
2018 Mar 4–2018 Mar 10	6,375	692	40.2
2018 Mar 11–2018 Mar 17	5,655	430	45.3

GP, general practitioners, ILI, influenza-like illness.
